# Identification of Poly(ethylene terephthalate) Nanoplastics in Commercially Bottled Drinking Water Using Surface-Enhanced Raman Spectroscopy

**DOI:** 10.1021/acs.est.3c00842

**Published:** 2023-05-23

**Authors:** Junjie Zhang, Miao Peng, Enkui Lian, Lu Xia, Alexandros G. Asimakopoulos, Sihai Luo, Lei Wang

**Affiliations:** † Department of Chemistry, Norwegian University of Science and Technology (NTNU), 7491 Trondheim, Norway; ‡ Laboratory of Environmental Toxicology and Aquatic Ecology, Faculty of Bioscience Engineering, Ghent University, Coupure Links 653, 9000 Ghent, Belgium; § College of Environmental Science and Engineering, Nankai University, Tianjin 300350, China

**Keywords:** nanoplastics, surface-enhanced Raman spectroscopy, bottled drinking water, nanosphere lithography

## Abstract

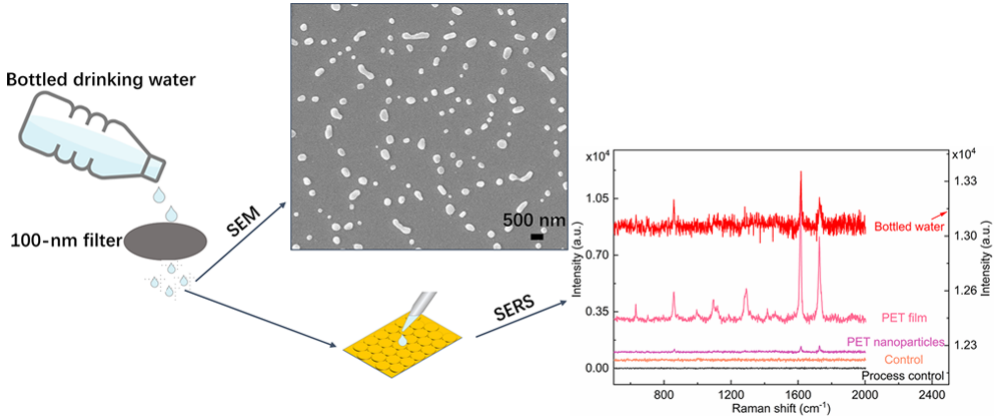

Micro/nanoplastics have emerged as global contaminants of serious concern to human and ecosystem health. However, identification and visualization of microplastics and particularly nanoplastics have remained elusive due to the lack of feasible and reliable analytical approaches, particularly for trace nanoplastics. Here, an efficient surface-enhanced Raman spectroscopy (SERS)-active substrate with triangular cavity arrays is reported. The fabricated substrate exhibited high SERS performance for standard polystyrene (PS) nanoplastic detection with size down to 50 nm and a detection limit of 0.001% (1.5 × 10^11^ particles/mL). Poly(ethylene terephthalate) (PET) nanoplastics collected from commercially bottled drinking water were detected with an average mean size of ∼88.2 nm. Furthermore, the concentration of the collected sample was estimated to be about 10^8^ particles/mL by nanoparticle tracking analysis (NTA), and the annual nanoplastic consumption of human beings through bottled drinking water was also estimated to be about 10^14^ particles, assuming water consumption of 2 L/day for adults. The facile and highly sensitive SERS substrate provides more possibilities for detecting trace nanoplastics in an aquatic environment with high sensitivity and reliability.

## Abstract 2

This study reports an efficient SERS substrate that can be used to detect nanoplastics in bottled drinking water.

## Introduction

Micro/nanoplastics, as emerging environmental contaminants, have gained attention in recent years due to their ubiquitous presence and the threat they pose to ecosystems, aquatic organisms, and human health.^1−5^ In particular, nanoplastics (<1 μm), due to their significantly small size, can be distributed in the ecosystem easier than microplastics (<5 mm).^6−8^ However, nanoplastics are not thoroughly investigated compared to microplastics due to the lack of effective methods for detecting and characterizing them at the sub-micrometer scale.^9−12^

Various techniques for detection of micro/nanoplastics, such as Fourier transform infrared (FTIR) spectroscopy,^4,12−14^ Raman spectroscopy,^12,15−18^ pyrolysis gas chromatography/mass spectrometry (GC-MS),^19−21^ liquid chromatography–tandem mass spectrometry (LC-MS/MS),^22,23^ transmission and scanning electron microscopy (TEM and SEM),^10,12,24,25^ fluorescence microscopy,^26−29^ dynamic light scattering (DLS),^30^ and nanoparticle tracking analysis (NTA),^31,32^ have been employed. Among these methods, FTIR and normal Raman methods were found not suitable for the detection of nanoplastics due to their low spatial resolution (approximately 10 and 1 μm, respectively) and also due to sample fluorescence interference. TEM and SEM were unable to determine the chemical profile of plastics. Although LC-MS/MS and GC-MS can identify chemical fingerprints of plastics with high sensitivity, the instruments are unable to identify individual nanoparticles and can damage the samples. Therefore, a simple and reliable method for efficient, rapid detection and identification of nanoplastics in the environment is urgently required.

Recently, surface-enhanced Raman spectroscopy (SERS) has received considerable attention in detecting environmental chemical hazards due to its high sensitivity, rapid response time in analysis, and good selectivity based on the unique fingerprints in Raman spectra of the target analytes.^33−36^ SERS has two main classes of enhancing media: metallic nanoparticles and solid substrates with roughed or nanostructured surfaces.^37^ Joo et al. reported a highly sensitive SERS substrate based on nanoparticle-embedded anodized aluminum oxide (AAO) arrays for the detection of nanoplastics (400 nm).^38^ Zhou et al. facilitated the detection of polystyrene (PS) nanoplastics (∼50 nm) using SERS-active silver nanoparticles.^39^ Nevertheless, these studies demonstrated low repeatability in reproducing the uniform morphology of assembled nanoparticles. Alternatively, to reply on metallic nanoparticles, large SERS enhancements were also observed from nanoplastics positioned inside the solid substrates. Chang et al. fabricated a SERS-active substrate composed of self-assembled SiO_2_ sputtered with silver films (SiO_2_ PC@Ag), which can test PS nanoplastics down to 200 nm, but it was not applicable for real environment sample detection.^40^ Zhang et al. demonstrated the possibility of detecting nanoplastics (360 nm) extracted from air using an expensive, lithographically fabricated SERS substrate (Klarite).^7^ Although these advances are used in identifying nanoplastics using SERS-active substrates, only a few are applicable for detecting real environmental samples.^41^ In addition, it is also highly desirable to build an integrated platform that combines low-cost, large-area fabrication of efficient SERS-active substrates toward actual environmental monitoring.

In this work, a simple, low-cost approach was proposed by using reliable and high-throughput fabrication of gold triangular cavity array (TCA) as a SERS-active substrate. The resulting triangular cavity arrays were successfully used to detect PS nanoplastics with a size of 50 nm and a detection limit of 0.001%. To further seek the practical application of this substrate, poly(ethylene terephthalate) (PET) nanoplastics collected from commercially bottled drinking water were also demonstrated. The low-cost, highly sensitive triangular cavity array substrate provided an efficient and rapid SERS-based strategy for nanoplastic detection in mineral water.

## Materials and Methods

### Materials

In all experiments, distilled water (18.2 MΩ·cm) was obtained from a Millipore filtration system. PS nanosphere aqueous suspensions (200, 500, 1000 nm, weight percent: 10% (w/v)) were purchased from Sigma-Aldrich (Darmstadt, Germany). PS nanosphere (50 nm, weight percent: 1% (w/v)) aqueous suspension was purchased from Phosphorex (Hopkinton, MA). Epoxy Norland NOA61 was purchased from Thorlabs (Newton, New Jersey). Gold (99.99%) and silver (99.99%) used for deposition were purchased from Kurt J. Lesker (Dresden, Germany). Trifluoroacetic acid (TFA, 99%) was purchased from Alfa Aesar (Haverhill, MA). Sodium dodecyl sulfate (SDS, 98%) was purchased from Acros Organics (Geel, Belgium). Acetone and absolute ethanol (99.5%, VWR chemicals, Oslo, Norway) were used as received without further purification.

### PET Nanoparticle Preparation and Characterization

The PET nanoparticles were prepared according to previously published studies.^42,43^ Briefly, 1 g of PET plastic debris, which was obtained from a drinking water bottle, was dissolved in 10 mL of a 90% (v/v) TFA/water mixture at 50 °C with stirring. After the solution was kept overnight, 10 mL of a TFA/water mixture (2/8, v/v) was added under vigorous stirring and kept stirred for 2 h, and then stored overnight. The solution was centrifuged at 2500 *g* for 1 h, and the supernatant was removed. The precipitate was resuspended in 1 L of 0.5% SDS and then ultrasonicated and settled in a cylinder for 1 h. The upper fraction in the cylinder was collected and characterized by atomic force microscopy (AFM) and NTA, as shown in Figure S1 in the Supporting Information.

### Fabrication of Gold Triangular Cavity Array Substrates

A piece of a wafer was cleaned with acetone, ethanol, and deionized water and dried by a gentle N_2(g)_ stream, followed by oxygen plasma for 1 min (100 W, oxygen flow rate: 5 mL/min). Thereafter, a 0.5 μL droplet of polystyrene (PS, 500 nm) nanospheres diluted at 2:1 (v/v) Milli-Q water/ethanol was placed onto a glass within a 1 cm diameter poly(dimethylsiloxane) (PDMS)-defined well and dried at ambient conditions to form a monolayer of close-packed PS nanospheres. Then, 50 nm thick silver was deposited onto the templated substrate by e-beam evaporation (e-beam evaporator and sputter AJA, AJA International Inc.) at 2 Å/s under 5 × 10^–7^ mbar. Then, the PS nanospheres were removed by a 3 M scotch tape, leaving behind the silver triangle arrays. Next, another layer of 150 nm gold was deposited over the entire substrate by e-beam evaporation at 2 Å/s under 5 × 10^–7^ mbar. An epoxy of a NOA61 film was drop-cast on top of the substrate, allowed to cure under UV (5 min) exposure, and then peeled away manually, transferring silver and gold side-by-side on the cured epoxy. In the final step, silver was removed by chemically etching (a mixture of an aqueous solution of HNO_3_ (70%)/HCl (37%)/H_2_O = 1:1:1) for 2 min, leaving gold triangular cavity arrays.

### Preparation of Samples Extracted from Commercially Bottled Drinking Water

Four brands of bottled drinking water (bottles were made of PET) were purchased from a local supermarket in Trondheim, Norway. A vacuum filtration device was used to filter the water (100 mL) through glass fiber filter membranes with pore diameters of 0.1 μm (100 nm, Xingyacailiao, China), and the filtrate was collected. A glass pipette was used to aspirate about 10 mL of the filtrate to an amber glass sample bottle for further analysis. Glass instruments were used during the experiment. Except for the Millipore Glass Base, glassware and the glass fiber filter membrane were baked in an oven at 500 °C for 4 h prior to use. The Millipore glass base was rinsed with 1 M hydrochloric acid, 1 M potassium hydroxide, and dichloromethane, and the suction filtration experiment was carried out in a fume hood. The procedural blank was performed using deionized water instead of the actual sample.

### Raman Spectra Measurements

Raman spectra were obtained on a Renishaw confocal micro-Raman spectrometer with a laser excitation wavelength of 532 nm. The laser beam was focused onto the sample through an 50× objective lens with the selected 1 mW laser power with a 10 s acquisition time. The Raman mapping images were recorded over a scan area of 2 μm × 2 μm at a step size of 200 nm for PS and a scan area of 3 μm × 3 μm at a step size of 300 nm for PET. All of the Raman spectra were baseline-corrected via Wire software (dedicated to the Raman spectrometer) to remove background noises in this study. To prepare the samples for Raman determination, a portion of 0.2 μL of aqueous PS with different sizes (50, 200, 500, and 1000 nm) was drop-cast onto gold triangular cavity array substrates and dried under ambient conditions. Likewise, 0.2 μL of filtered bottled water was drop-casted onto the gold TCA substrates and dried under ambient conditions, and in all cases, Raman spectra were measured from a minimum of three randomly chosen sample areas on a given substrate.

### Imaging

Samples for SEM imaging were prepared by drop-casting 0.2 μL of the filtrate on a silicon wafer and drying under ambient conditions. SEM images were recorded on an electron microscope (FEI APREO) using an electron-beam voltage of 10 kV and a current of 13 pA.

### Nanoparticle Tracking Analysis

Nanosight LM10 (NanoSight, Wiltshire, U.K.) was used for nanoparticle tracking analysis (NTA). A 0.5 mL solution of an analyte was introduced to the optical viewing cell, and 60 s videos were recorded for each sample. Nanoparticles were counted and analyzed by NTA image analysis software version 3.3, giving the attenuated curves of particle size distribution and number concentration.

## Results and Discussion

### Fabrication and Characterization of the Gold Triangular Cavity Array Substrate

**Figure 1:**
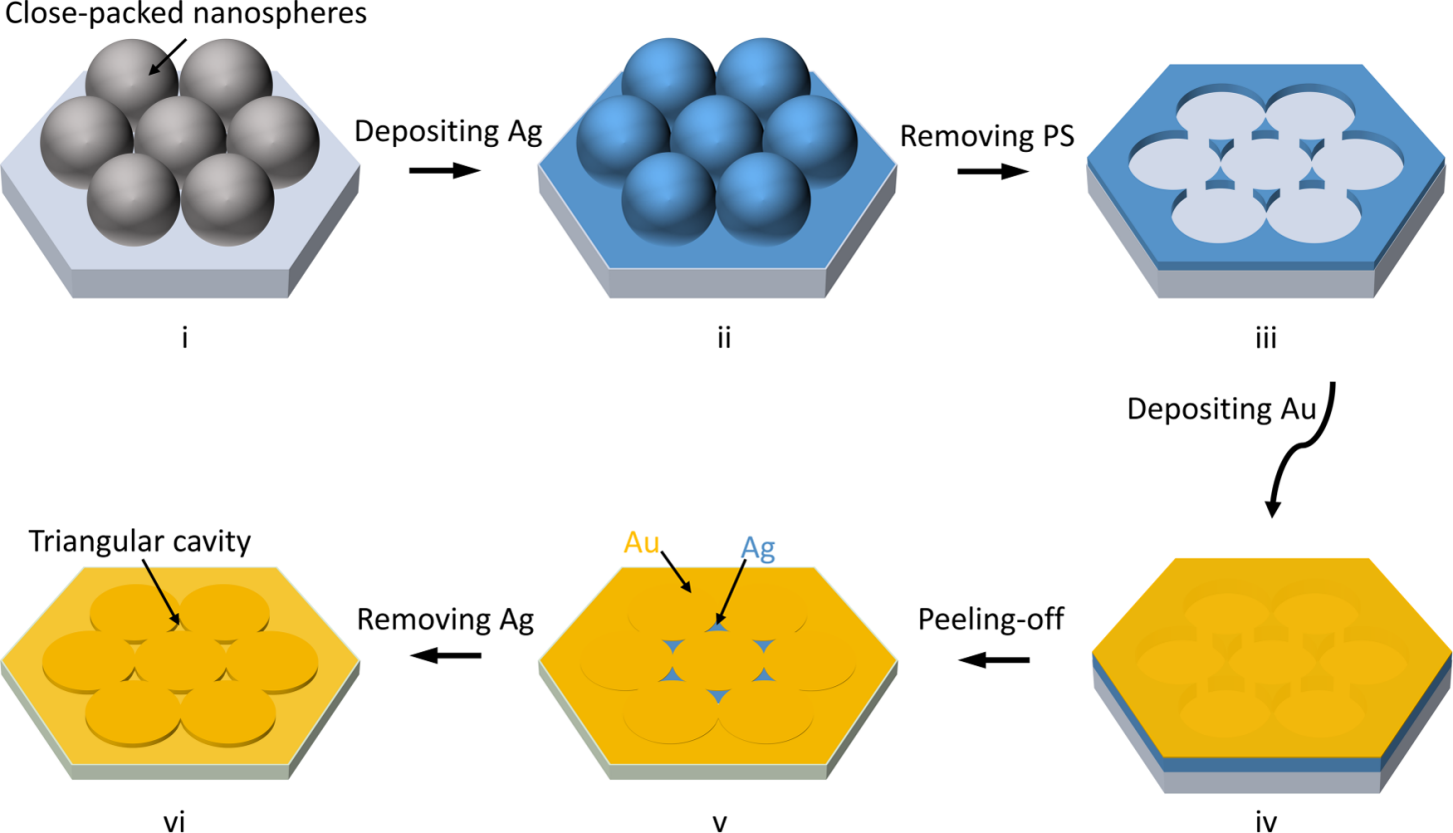
Schematic illustration of the fabrication process for the triangular cavity array (TCA). First, close-packed polystyrene (PS) nanospheres are self-assembled on a silicon substrate (i). A thin silver (Ag) film is deposited over the nanospheres (ii), which are then tape-stripped away, leaving Ag nanotriangle arrays (iii). A gold (Au) film is then deposited over the entire substrate (iv). An adhesive epoxy is applied on the top of Au and then peeled off, transferring two metals Ag and Au, sitting in a complementary arrangement side-by-side on epoxy (v). Simply removing the Ag parts using chemical etching revealed gold TCA, as shown in (vi).

**Figure 2:**
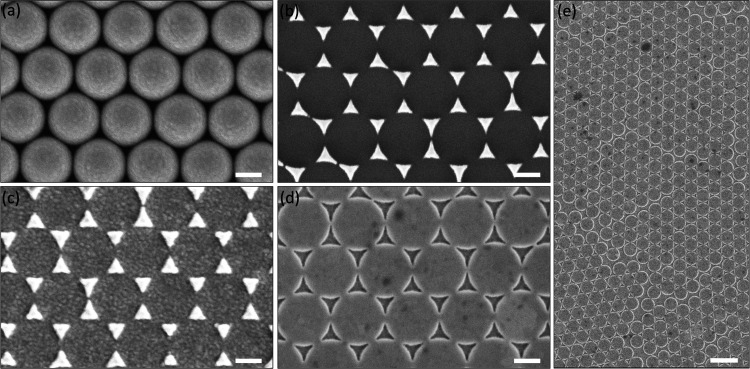
Scanning electron micrographs (SEMs) of the corresponding processing steps in Figure 1 to fabricate the gold TCA substrate. (a) Close-packed PS nanospheres that correspond to step i in Figure 1. (b) Ag triangle arrays after removing PS nanospheres that correspond to step iii in Figure 1. (c) Top-view morphology after depositing a Au layer that corresponds to step iv in Figure 1. (d) Au TCA after removing Ag parts that correspond to step vi in Figure 1. The scale bar in (a)–(d) is 250 nm. (e) Patterned gold TCA over a large area; the scale bar in (e) is 1 μm.

The fabrication process for making a gold TCA substrate is shown in Figure 1. First, a monolayer of close-packed 500 nm PS nanospheres was assembled on a precleaned silicon substrate, as shown in Figure 1-i. Then, a thin silver film (50 nm, Ag) was deposited over the nanospheres (Figure 1-ii), and the nanospheres were removed by tape-stripping, leaving Ag nanotriangle arrays (see Figure 1-iii). Next, a thick gold film (150 nm, Au, see Figure 1-iv) was deposited over the entire substrate. Subsequently, an adhesive epoxy was applied uniformly to the surface of Au, allowed to cure under UV exposure, and then peeled away, transferring two metals sitting on a complementary arrangement side-by-side on the peeled epoxy. Simply removing the Ag triangles using chemical etching revealed gold TCA, as demonstrated in Figure 1-vi. Figure 2a–e shows scanning electron micrographs (SEMs) of the corresponding processing steps for gold TCA. As shown in Figure 2e, well-defined nanotriangluar cavities (side length of 160 nm) were formed along the prenanosphere-templated nanotriangular edges of Ag over a large area. Such nanoscale sharp apex and edges of cavities can generate tremendous electromagnetic field enhancements and localization of incident light and thus are suitable for SERS analysis.^40,44−46^ To elucidate the mechanism and further optimization of a TCA substrate for SERS application, electric field distribution simulations were performed under illumination wavelengths of 532, 633, and 785 nm, as shown in Figure S2. The enhanced field is mostly concentrated in the apex and edges of the triangular cavities and is the strongest under the illumination of 532 nm. It indicates that the SERS enhancement is strongly dependent on the particle location relative to the triangular cavity. Therefore, the SERS enhancement is mainly attributed to electric field enhancement increased by the edges and apex of triangular cavities that the particles fall into or are close to.^7,18^

### Method Application for Standard PS Nanoplastics

**Figure 3:**
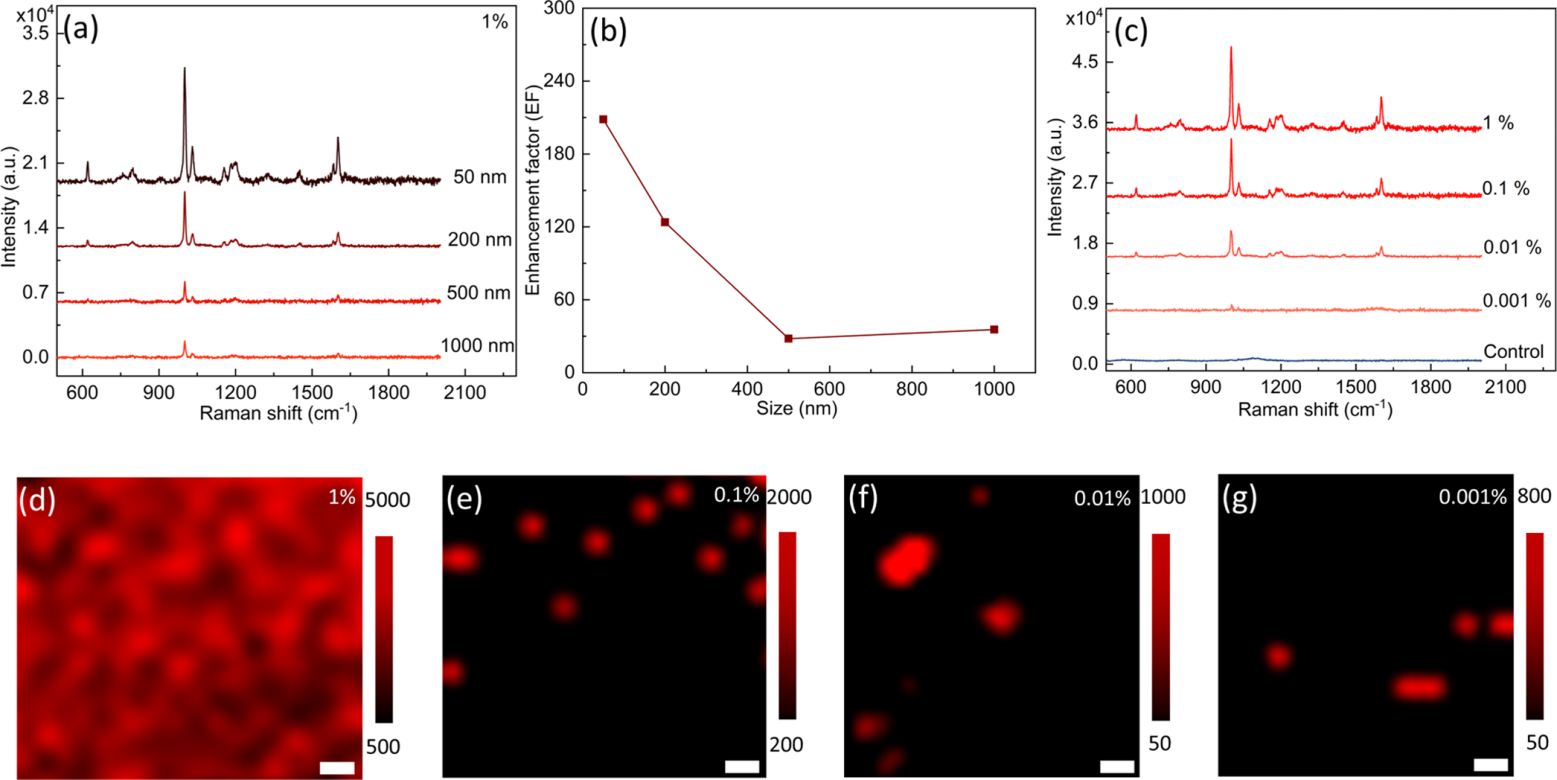
(a) Raman spectra of PS nanoplastics with different sizes on Au TCA substrates at a concentration of 1%. (b) Enhancement factor (EF) as a function of PS size. (c) Raman spectra of 50 nm PS nanoplastics with concentrations varying from 1 to 0.001% on TCA substrates and on a plain glass substrate at a concentration of 1% (control line). (d–g) Raman mapping images of 50 nm PS nanoplastics on Au TCA substrates with different concentrations from 1 to 0.001%. The scale bar in (d)–(g) is 200 nm.

The Raman spectra of PS particles with different sizes at a concentration of 1% located on the as-fabricated gold TCA substrates are shown in Figure 3a. The Raman signal of PS particles is clearly detectable according to the two distinguished peaks at 1003 and 1033 cm^–1^, which are attributed to the ring-mode vibrations of a monosubstituted aromatic compound C–C and C–H in PS.^7^ The 50 nm PS presented a higher enhanced Raman signal compared to 200, 500, and 1000 nm, indicating that the SERS effect is dependent on the size of the nanoplastics relative to the pitch size of TCA. The enhancement factor (EF) of the TCA substrate was calculated to evaluate the enhancement effect (see Table S1 in the Supporting Information), and the peak intensity of the PS nanoplastics at 1003 cm^–1^ was selected to calculate the EF, as shown in Figure 3b. The EF of PS nanoplastics decreased as particle sizes increased, and the EF of 50 nm PS nanoplastics reached the highest value of 208.5. One possible explanation for the highest SERS intensity for 50 nm PS may be due to the higher probability of smaller PS particles to be located inside the nanotriangular cavity regions per unit area and, therefore, benefit more from the electric field enhancement (see Figure S3). Therefore, the enhancement effect on nanoplastics depends on both position and size, which agrees well with previous studies.^7,18^ The Raman spectra of 50 nm PS particles on the gold TCA substrates, with concentrations varying from 1 to 0.001% (w/v), and on glass substrate at a concentration of 1%, are shown in Figure 3c. No Raman signal of PS was detected on a plain glass substrate, while highly enhanced characteristic Raman signals of PS were observed on a gold TCA substrate at a concentration of 1%. The Raman spectra clearly demonstrated the characteristic peaks of PS for different concentrations from 0.1 to 0.001%. Distinguished signals of PS with a concentration of 0.001% at 1003 and 1030 cm^–1^ were still detectable (the signal-to-noise ratios were 88 and 86, respectively, calculated by the mean of the peak height divided by the standard deviation of the background (1900–2000 cm^–1^)^47^) (see Figure 3c). Compared with the Klarite substrate (commercial SERS substrate), which has been used to detect nanoplastics with a size down to 360 nm,^7^ our method indicates that nanoplastics with a size of 50 nm could be detected. Raman mapping images of PS nanoplastics with different concentrations on the TCA substrate were also recorded, as shown in Figure 3d–g. Overall, 1% of 50 nm PS nanoplastics was indistinguishable in the mapping image due to a large number of particles (see Figure 3d). The number of particles shown in the mapping images decreased as the concentration decreased from 1 to 0.001% (1.5 × 10^11^ particles/mL), indicating that the TCA substrate, as an efficient SERS-active platform, has the potential to quantify nanoplastics by counting and can be used to identify and visualize nanoplastics.

### Method Application in Commercially Bottled Drinking Water

**Figure 4:**
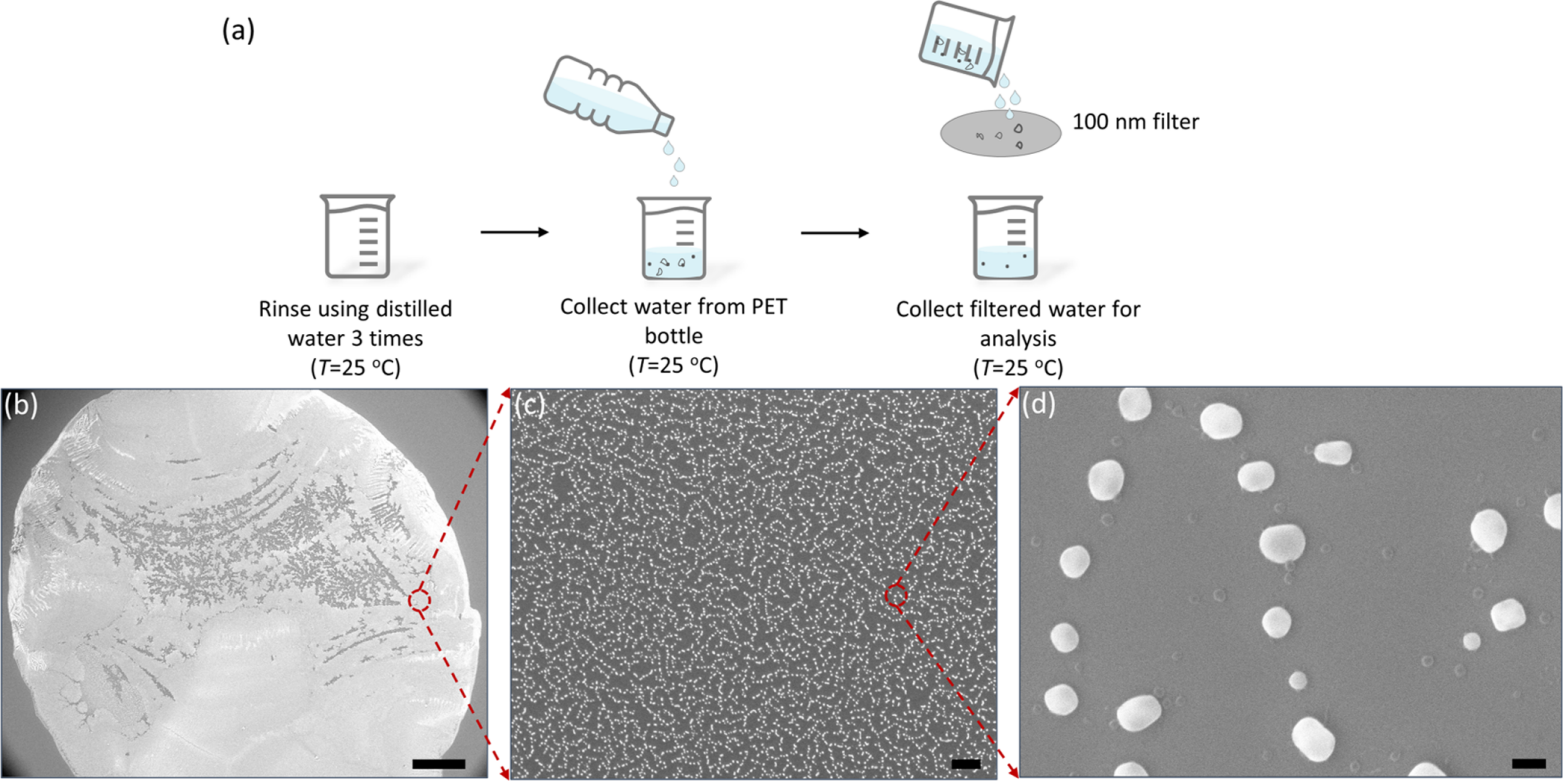
(a) Schematic of sample preparation from commercially bottled drinking water. (b–d) SEM images of an extracted sample that was drop-cast on a silicon wafer after drying under ambient conditions. Scale bar: (b) 300 μm, (c) 5 μm, and (d) 200 nm.

**Figure 5:**
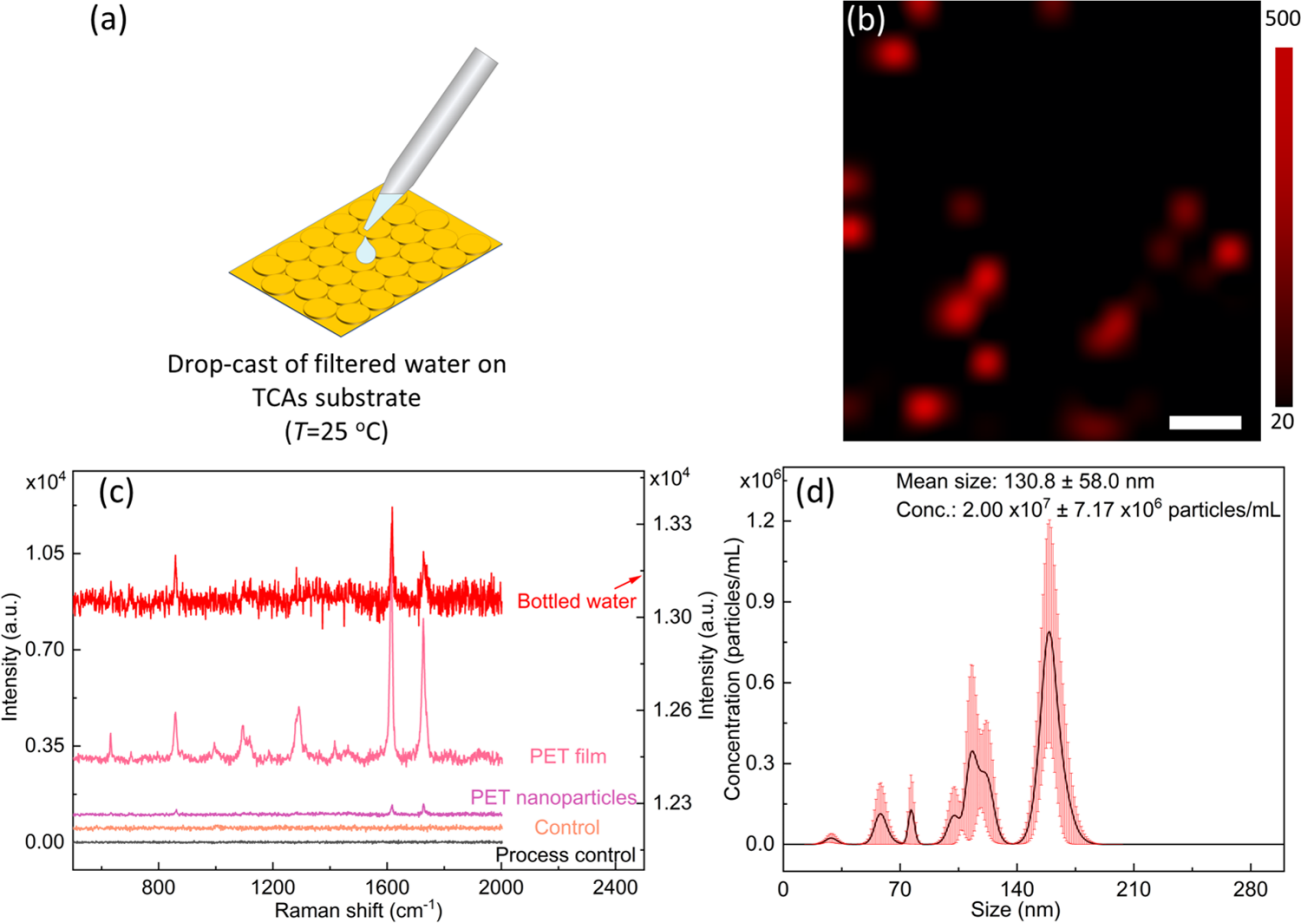
(a) Schematic of sample preparation from bottled drinking water. (b) Raman mapping image of the sample extracted from bottled drinking water on the TCA substrate. Scale bar: 500 nm. (c) Raman spectra of the sample extracted from bottled drinking water on the TCA substrate (red line), the plain glass substrate (brown line), and the PET film (purple line). (d) Finite track length adjustment (FTLA) concentration/size image for NTA of the sample extracted from bottled drinking water on the TCA substrate: the indicated mean size of nanoplastics is ca. 130.8 ± 58.0 nm.

Potential exposure of human beings to nanoplastics from commercially bottled drinking water has attracted significant attention due to a higher risk to human health than microplastics.^48,49^ The procedure of sample preparation from bottled water is shown in Figure 4a, and SEM images of the nanoplastics extracted from bottled drinking water made of PET are shown in Figure 4b–d. The SEM images of the procedure blank using deionized water are shown in Figure S4, indicating that no external contaminants were introduced during the entire process. In addition, the reusability of the substrates was tested three times using PET nanoparticles (see Figure S5), and it showed good reusability. Tremendous nanoparticles with a mean size of ∼122 nm extracted from SEM image directly (see Figure S6) were observed after 100 nm filtration of collected bottled water, as shown in Figure 4c,d. The practical applicability of TCA was further validated by identifying these nanoplastics extracted from bottled drinking water, as shown in Figure 5. Both Raman spectra and mapping were recorded. The distinguishable particle size, i.e., ∼150 nm and less, in the mapping image matches well with that observed in SEM images (see Figures 4d and 5b,d). The characteristic peaks at 1620 and 1760 cm^–1^ corresponding to the C–O stretching and C=O stretching vibration,^7^ respectively, were found to be comparable with both the PET film and synthesized PET nanoparticle standard spectra presented in Figure 5c as well, suggesting the presence of PET nanoplastics in bottled drinking water as there were no signals in a procedure blank. The signal-to-noise ratios of the characteristic peaks at 1620 and 1760 cm^–1^ from the filtrate of bottled water samples were 6.2 and 4.2, respectively (calculated by the same method for PS).^47^ Furthermore, the number of nanoplastics released in bottled drinking water was determined by NTA, as shown in Figure 5d. The instrument software reported that the mean size of particles was 88.2 ± 50 nm with a concentration of (1.66 × 10^8^) ± (2.33 × 10^7^) particles/mL, which was calculated by the particle numbers and particle sizes of the three replicates (Table S2, see extra two replicates in Figure S7), indicating that tremendous nanoplastics were released into the water compared with microplastics of around 10^–1^–10^4^ particles/mL.^50^ The main reason for the particle size above 100 nm comes from the systematic deviation using NTA. The mean size extracted from NTA is based on the assumption that all particles are regarded as standard spherical particles. If a particle has a rod-like shape, the measured size may be larger than its actual size on average. In addition, the aggregation of the nanoparticles after filtration cannot be ignored even if the samples are ultrasonicated before analysis. In addition, three other bottled water samples from different brands were also investigated, as shown in Figure S8, further confirming the presence of a large amount of nanoplastics in bottled drinking water. Notably, Zangmeister et al. recently reported that single-use food-grade nylon bags and hot beverage cups lined with low-density polyethylene (LDPE) could also release large amounts of nanoplastics of >10^9^ particles/mL when exposed to hot water (100 °C).^31^ Finally, the annual nanoplastic consumption by human beings through bottled water was estimated to be around (5.4 × 10^13^)–(1.3 × 10^14^) particles, considering the water consumption of 2 and 1 L/day for adults and children, respectively, which was significantly higher than the annual microplastic consumption (around (7.4 × 10^4^)–(1.2 ×10^5^) particles/year) reported in a previous study.^51^

### Environmental Implication

A simple, low-cost, and high-throughput patterning methodology was demonstrated to fabricate a Au TCA substrate for efficient SERS analysis of PS and PET nanoplastics in water. The fabricated Au TCA substrate demonstrates high sensitivity in detecting PS nanoplastics with a size down to 50 nm and a detection limit of 0.001%. The substrate was also used to identify PET nanoplastics collected from commercially bottled drinking water with a size up to ∼200 nm. Furthermore, the concentration of the collected sample was determined to be 10^8^ particles/mL by NTA. However, the composition of nanoplastics that existed in commercially bottled drinking water is complex, not only from the pollution of the bottle but also from the pollution of the water source and the packaging process. Thus, the detection of trace nanoplastic composite pollutants remains a challenge for the TCA substrate.

## Supporting Information Available

Atomic force microscopy image and concentration/size image for NTA of synthesized PET (Figure S1), simulated electric field distribution of the Au TCA substrate at different excitation wavelengths (Figure S2), EF of PS nanoplastics with different sizes (Table S1), schematic of a spherical particle and SEM images of 50 nm PS particles on triangular cavity array (Figure S3), SEM images of the drinking bottled water samples and procedural blank (Figure S4), Raman spectra of synthesized PET nanoparticles deposited on the nanotriangular cavity array substrate with three recyling (Figure S5), histogram of nanoplastic sizes extracted from Figure 4c (Figure S6), Raman spectra and concentration/size image for NTA of two samples from the same bottled drinking water sample (Figure S7), nanoparticle sizes and concentrations of the three replicates and its mean values (Table S2), and Raman spectra and concentration/size image for NTA of the sample extracted from three other different brands’ bottled drinking water (Figure S8) (PDF)
