# Supplementary material for: Identification of Poly(ethylene terephthalate) Nanoplastics in Commercially Bottled Drinking Water Using Surface-Enhanced Raman Spectroscopy
Source: Environ Sci Technol. 2023 May 23;57(22):8365–72. doi: 10.1021/acs.est.3c00842 (PMC10249414; doi:10.1021/acs.est.3c00842)
Supplement: Supplementary file 1 — es3c00842_si_001.pdf [file es3c00842_si_001.pdf]

Identification of polyethylene terephthalate nanoplastics in commercially bottled drinking water using surface-enhanced Raman spectroscopy

Junjie Zhang[1], Miao Peng[2], Enkui Lian[1], Lu Xia,[1] Alexandros G. Asimakopoulos[1], Sihai Luo[1*], Lei Wang[3*]*

[1]Department of Chemistry, Norwegian University of Science and Technology (NTNU), 7491 Trondheim, Norway

[2]Laboratory of Environmental Toxicology and Aquatic Ecology, Faculty of Bioscience Engineering, Ghent University, Coupure Links 653, 9000 Ghent, Belgium

[3]College of Environmental Science and Engineering, Nankai University, Tianjin, 300350, China

[*]Corresponding authors: Alexandros G. Asimakopoulos (alexandros.asimakopoulos@ntnu.no), Sihai Luo (sihai.luo@ntnu.no), Lei Wang (wang2007@nankai.edu.cn)

Content Summary

Number of Pages: 11

Number of Tables: 2

Number of Figures: 8

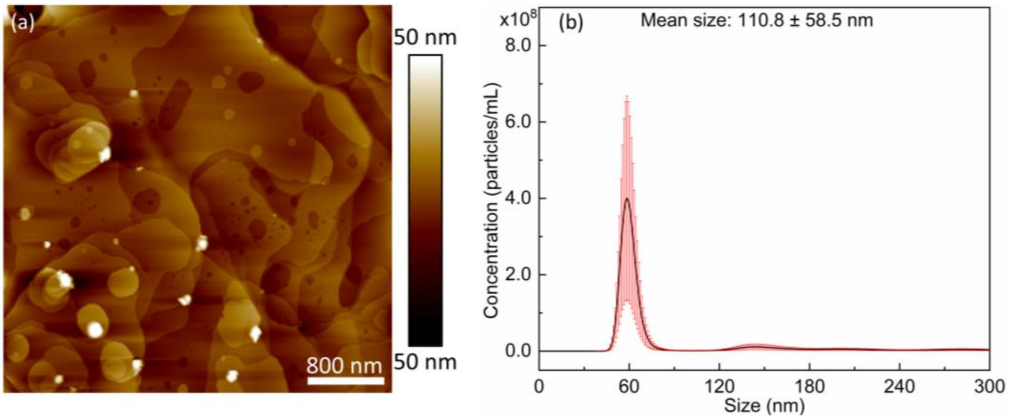

Figure S1 (a) Atomic force microscopy image of PET nanoparticles as reference that synthesised by following literature[1]. (b) Associated finite track length adjustment (FTLA) concentration/size image for NTA of synthesized PET nanoparticles, indicating the mean size is about 110 nm.

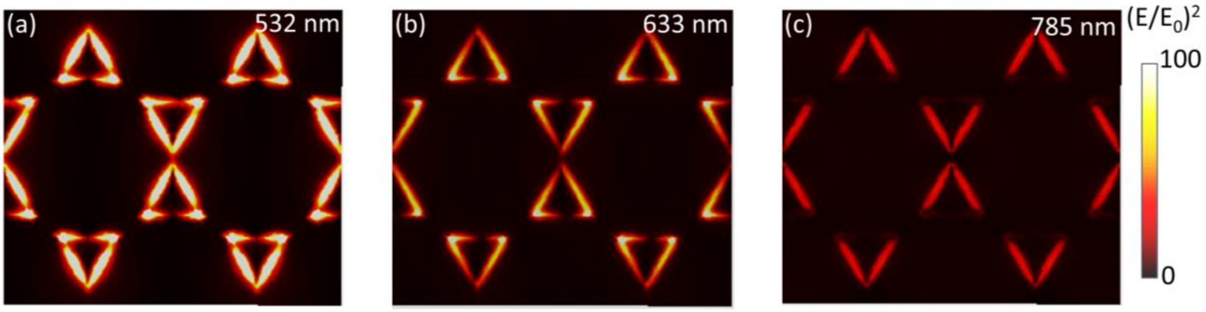

Figure S2 Simulated electric field distribution of Au TCAs substrate at different excitation wavelength

Determination of the Raman scattering enhancement factor (EF) relative to a plain glass.

EF was calculated according to the following equation[2,3]:

$$EF = \frac{I_{SERS}/N_{SERS}}{I_{NRS}/N_{NRS}},$$

Where I_{SERS} and I_{NRS} are defined as peak intensities of PS at 1003 cm^{-1} detected by the SERS substrate (TCAs) and non-SERS substrate (plain glass), respectively; N_{SERS} and N_{NRS} refer to the number of analytes that contribute to the intensity of SERS and non-SERS Raman peak intensities, repsectively. All Raman spectra were obtained using identical aquisition parameters, see experimental section. In addition, all samples were prepared with consistent concentrations (1%) of PS spheres. Hence, the number of contributing particles (Ratio of $N_{SERS}$and $N_{NRS}$is fixed as 1) for a given particle size was considered to be constant between SERS and non-SERS samples. The Raman intensity peak height was measured after removing the baseline spectrum of the substrate. The calculated EF of PS nanoplastics with different sizes were shown in Table S1 below.

Table S1 EF of PS nanoplastics with different sizes based on the Raman intensities at 1003 cm^{-1} of PS.

PS size/nm	I_{SERS}	I_{NRS}	EF
50	12302	59	208.5
200	5698	46	123.8
500	2220	79	28
1000	1773	50	35.46

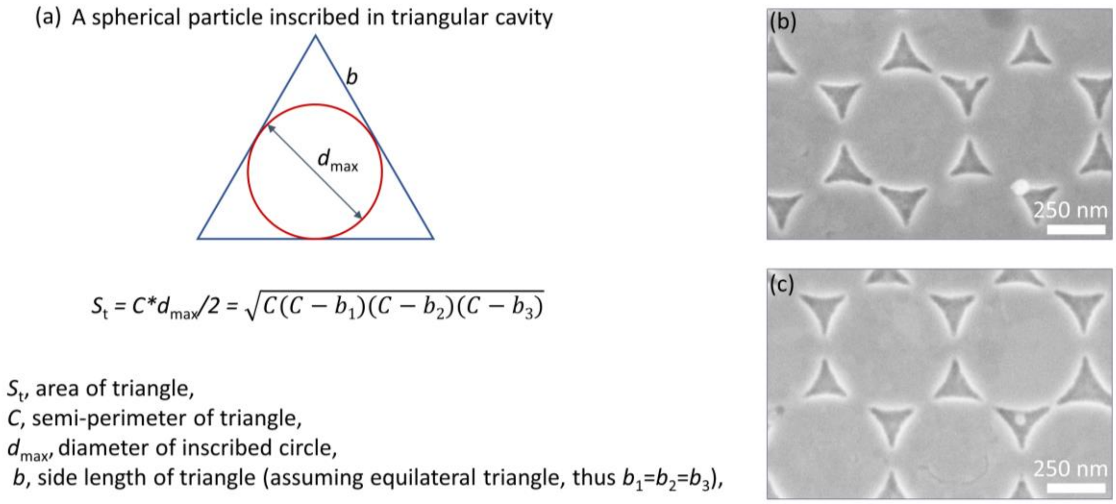

Figure S3 (a) Schematic of a spherical particle inscribed in triangular cavity assuming that trangular cavity is equilateral triangle, giving maximum diameter of a spherical particle inscribed about 92 nm if side length of triangle is 160 nm. (b, c) SEM images of 50 nm PS particles deposited on triangular cavity arrays, which were either trapped in cavity or close to apex or edges of cavity.

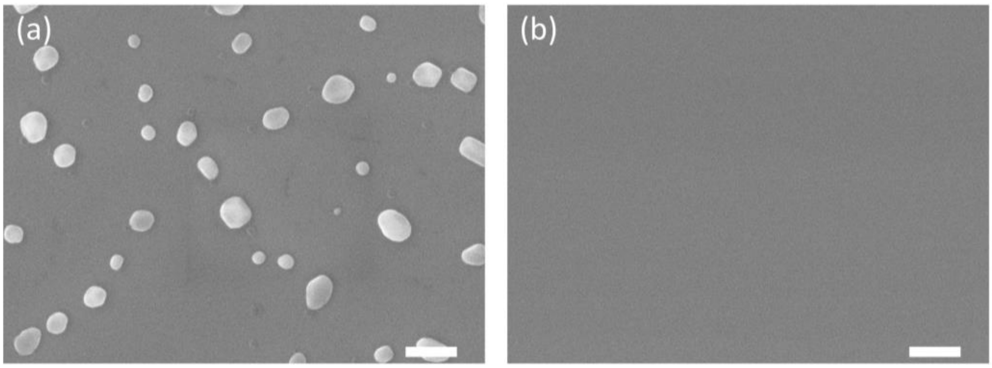

Figure S4 Scanning electron microscopy images of samples collected from bottled drinking water (a) and procedure blank (b). Scale bar: 400 nm.

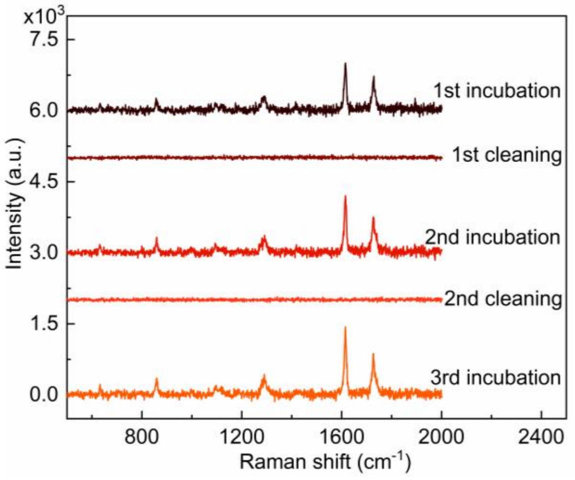

Figure S5 Raman spectra of syntehsied PET nanoparticles deposited on nanotriangular cavity arrays substrate with 3 recyling, indicating the substrate have good reusability. Substrate was cleaned with extensively ethanol, and distilled water, and then dried with Nitrogen gas gun after each incubation in PET nanoparticles solution.

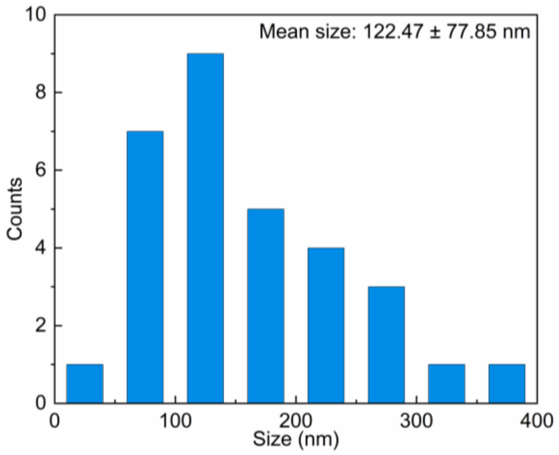

Figure S6 Histogram of nanoplastics size extracted from Figure 4c, showing the mean size of ~122 nm.

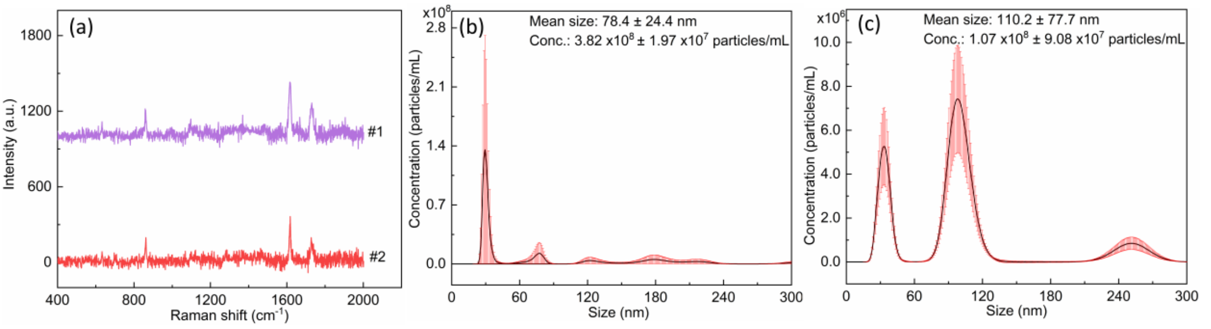

Figure S7 (a) Raman spectra of 2 replicated samples from a bottled drinking water. (b-c) Associated Associated finite track length adjustment (FTLA) concentration/size image for NTA of sample extracted from 2 samples collected from a bottled drinking water.

Table S2 Nanoparticle sizes and concentrations of the three replicates and its mean values[a]

	Size (nm)	Concentration (particles/mL)
Figure 5d	130.8±58	$2.00\times10^{7} \pm 7.17\times10^{6}$
Figure S7b	78.4±24.4	$3.82\times10^{8} \pm 1.97\times10^{7}$
Figure S7c	110.2±77.7	$1.07\times10^{8} \pm 3.54\times10^{7}$
Mean	88.2±50	$1.66\times10^{8} \pm 2.33\times10^{7}$

[a] $mean_{size} = \frac{1}{2}(\frac{\sum_i[(x_i-\sigma_i)\times c_i]}{\sum_i c_i} + \frac{\sum_i[(x_i+\sigma_i)\times c_i]}{\sum_i c_i}) \pm std\left(\frac{\sum_i[(x_i-\sigma_i)\times c_i]}{\sum_i c_i}, \frac{\sum_i[(x_i+\sigma_i)\times c_i]}{\sum_i c_i}\right)$

$\mathrm{mean}_{concentration} = \frac{1}{2}\left(\frac{\sum_i(c_i-\alpha_i)}{3} + \frac{\sum_i(c_i+\alpha_i)}{3}\right) \pm std\left(\frac{\sum_i(c_i-\alpha_i)}{3} + \frac{\sum_i(c_i+\alpha_i)}{3}\right),$

The given equation involves various parameters for each sample, including the sample numbers (i), mean size (x), standard deviation (std) of particle sizes (σ), mean concentration (c), and standard deviation of concentrations (α). These parameters are calculated for three samples, as indicated by the inclusion of data from figure 5d, s7b, and s7c.

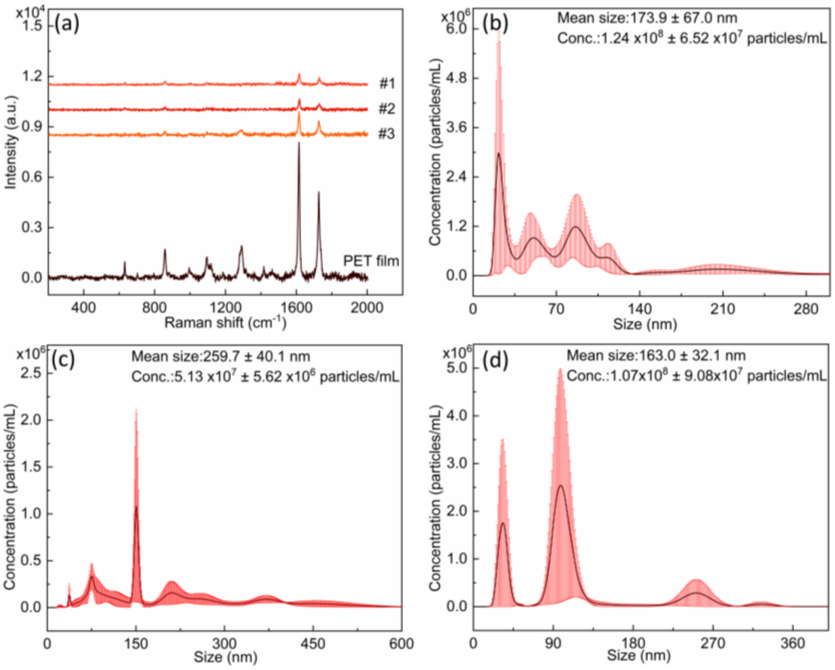

Figure S8 (a) Raman spectra of sample extracted from other 3 different brands bottled drinking water on TCAs substrate. (b-d) Associated finite track length adjustment (FTLA) concentration/size image for NTA of sample extracted from other 3 different brands bottled drinking water on triangular cavity arrays substrate.

References

(1) Rodríguez-Hernández, A. G.; Muñoz-Tabares, J. A.; Aguilar-Guzmán, J. C.; Vazquez-Duhalt, R. A Novel and Simple Method for Polyethylene Terephthalate (PET) Nanoparticle Production. *Environ. Sci. Nano* **2019**, *6* (7), 2031–2036. https://doi.org/10.1039/c9en00365g.

(2) Yang, Q.; Zhang, S.; Su, J.; Li, S.; Lv, X.; Chen, J.; Lai, Y.; Zhan, J. Identification of Trace Polystyrene Nanoplastics Down to 50 Nm by the Hyphenated Method of Filtration and Surface-Enhanced Raman Spectroscopy Based on Silver Nanowire Membranes. **2022**. https://doi.org/10.1021/acs.est.2c02584.

(3) Xu, G.; Cheng, H.; Jones, R.; Feng, Y.; Gong, K.; Li, K.; Fang, X.; Tahir, M. A.; Valev, V. K.; Zhang, L. Surface-Enhanced Raman Spectroscopy Facilitates the Detection of Microplastics <1 Mm in the Environment. *Environ. Sci. Technol.* **2020**, *54* (24), 15594–15603. https://doi.org/10.1021/acs.est.0c02317.
